# Optimizing the MLC model parameters for IMRT in the RayStation treatment planning system

**DOI:** 10.1120/jacmp.v16i5.5548

**Published:** 2015-09-08

**Authors:** Shifeng Chen, Byong Yong Yi, Xiaocheng Yang, Huijun Xu, Karl L. Prado, Warren D. D'Souza

**Affiliations:** ^1^ Department of Radiation Oncology University of Maryland School of Medicine Baltimore MD USA

**Keywords:** treatment planning system, IMRT commissioning, MLC model, TG‐119

## Abstract

Unlike other commercial treatment planning systems (TPS) which model the rounded leaf end differently (such as the MLC dosimetric leaf gap (DLG) or rounded leaf‐tip radius), the RayStation TPS (RaySearch Laboratories, Stockholm, Sweden) models transmission through the rounded leaf end of the MLC with a step function, in which the radiation transmission through the leaf end is the square root of the average MLC transmission factor. We report on the optimization of MLC model parameters for the RayStation planning system. This (TPS) models the rounded leaf end of the MLC with the following parameters: leaf‐tip offset, leaf‐tip width, average transmission factor, and tongue and groove. We optimized the MLC model parameters for IMRT in the RayStation v. 4.0 planning system and for a Varian C‐series linac with a 120‐leaf Millennium MLC, and validated the model using measured data. The leaf‐tip offset is the geometric offset due to the rounded leaf‐end design and resulting divergence of the light/radiation field. The offset value is a function of the leaf‐tip position, and tabulated data are available from the vendor. The leaf‐tip width was iteratively evaluated by comparing computed and measured transverse dose profiles of MLC defined fields at $d_{\text{max}}$ in water. In‐water profile comparisons were also used to verify the MLC leaf position (leaf‐tip offset). The average transmission factor and leaf tongue‐and‐groove width were derived iteratively by maximizing the agreement between measurements and RayStation TPS calculations for five clinical IMRT QA plans. Plan verifications were performed by comparing MapCHECK2 measurements and Monte Carlo calculations. The MLC model was validated using five test IMRT cases from the AAPM Task Group 119 report. Absolute gamma analyses (3 mm/3% and 2 mm/2%) were applied. In addition, computed output factors for MLC‐defined small fields ($2\times 2,3\times 3,4\times 4,6\times 6\;{\text{cm}}^{2}$) of both 6 MV and 18 MV photons were compared to those independently measured by the Imaging and Radiation Oncology Core (IROC), Houston, TX. 6 MV and 18 MV models were both determined to have the same MLC parameters: leaf‐tip offset=0.3 cm,2.5% transmission, and leaf tongue‐and‐groove width=0.05 cm. IMRT QA analysis for five test cases in TG‐119 resulted in a 100% passing rate with 3 mm/3% gamma analysis for 6 MV, and >97.5% for 18 MV. The passing rate was >94.6% for 6 MV and >90.9% for 18 MV when the 2 mm/2% gamma analysis criteria was applied. These results compared favorably with those published in AAPM Task Group 119. The reported MLC model parameters serve as a reference for other users.

PACS number(s): 87.55.D, 87.56.nk

## I. INTRODUCTION

During the last two decades, intensity‐modulated radiation therapy (IMRT) has become a widely used treatment modality in radiation oncology. In IMRT, each treatment field consists of multiple segments, or beamlets, shaped with the computer‐controlled multileaf collimator (MLC). This delivery technique with multiple segments makes it possible to deliver highly conformal doses to the target. The delivery of radiation via many small beamlets makes it challenging to accurately calculate dose in the treatment planning system (TPS). This challenge arises due to the difficulty in modeling the MLC in the TPS. Molineu et al.^(1)^ analyzed 1139 results of the Radiological Physics Center's (RPC) (now renamed as IROC) anthropomorphic head and neck IMRT phantom irradiations performed by 763 institutions undergoing clinical trial credentialing from 2001 to 2011, and found that only 81.6% of irradiations passed the gamma analysis criteria of 7%/4 mm when the treatment plan and treatment delivery were compared. Only 69% of irradiations passed the gamma analysis criteria of 5%/4 mm. A major reason for this surprisingly low passing rate is attributed to inadequate TPS commissioning for IMRT. Several publications have reported on the significance of adequate MLC modeling in IMRT dose calculations. LoSasso et al.^(2)^ reported that 1 mm position error for a 1 cm wide field resulted in 10% dose error for sliding window based IMRT. Cadman et al.^(3)^ investigated the effects of transmission through MLC rounded leaf ends in the TPS for step‐and‐shoot IMRT plans, and reported that the calculated dose was underestimated by up to 12% without appropriate consideration of leaf‐end transmission. Lee et al.^(4)^ studied the effects of different static dosimetric leaf gaps (a method to model the MLC rounded leaf end) for intensity‐modulated radiosurgery and reported that large dose differences (up to 12.7%) were associated with different leaf gaps (i.e., different MLC model parameters).

Commissioning of the TPS for IMRT remains a challenge. Although the AAPM has published a guidance document pertaining to the clinical implementation of IMRT,^(5)^ there are no consensus guidelines for acceptance criteria in TPS commissioning. The AAPM Task Group (TG) Report 119^(6)^ has benchmarked IMRT QA results from five institutions for a suite of test cases, resulting in a baseline reference for the accuracy that may be achieved in IMRT commissioning.

One of the main contributors to degree of agreement between calculated dose in the TPS and measured dose is the accuracy with which the MLC is modeled during commissioning of the TPS. The focus of this work is the modeling of the MLC in the RayStation planning system (RaySearch Laboratories, Stockholm, Sweden). It models the rounded leaf‐end MLC with four parameters: leaf‐tip offset, leaf‐tip width, average transmission factor, and tongue and groove. The model is different from other commercial TPS in the following manner: a) the MLC leaf‐end transmission is modeled using the leaf‐tip width instead of the MLC dosimetric leaf gap (DLG)^2^, ^4^, ^7^ or rounded leaf‐tip radius;^8^, ^9^, ^10^ b) the MLC leaf radiation transmission is modeled using average transmission factor instead of intraleaf and interleaf transmission.^8^, ^10^ The MLC leaf‐tip width specifies the dimension of leaf end which has transmission factor of square root of average MLC radiation transmission. Although the MLC modeling for IMRT commissioning has been reported multiple times for different commercial TPS,^3^, ^7^, ^8^, ^9^, ^10^ to our knowledge this is the first report of a verification of a new MLC model in a commercial TPS that states the dosimetric accuracy achievable by the RayStation TPS. In this study, we report the optimal MLC model parameters for the Varian C‐series with the Millenium‐120 MLC. The MLC modeling parameters were validated with IMRT cases from AAPM TG‐119 and with additional clinical cases.

## II. MATERIALS AND METHODS

### A. Configuration of MLC parameters

Details of the Varian MLC design can be found in AAPM Task Group Report No. 72.^(11)^ The RayStation treatment planning system models the MLC using the following parameters: 1) leaf‐tip offset, 2) leaf‐tip width, 3) average transmission factor, and 4) tongue‐and‐groove width. All these parameters were optimized for the 6 MV beam, but adopted for 18 MV photon; the results showed good agreements, as shown below.

#### A.1 Leaf‐tip offset

**Figure 1:**
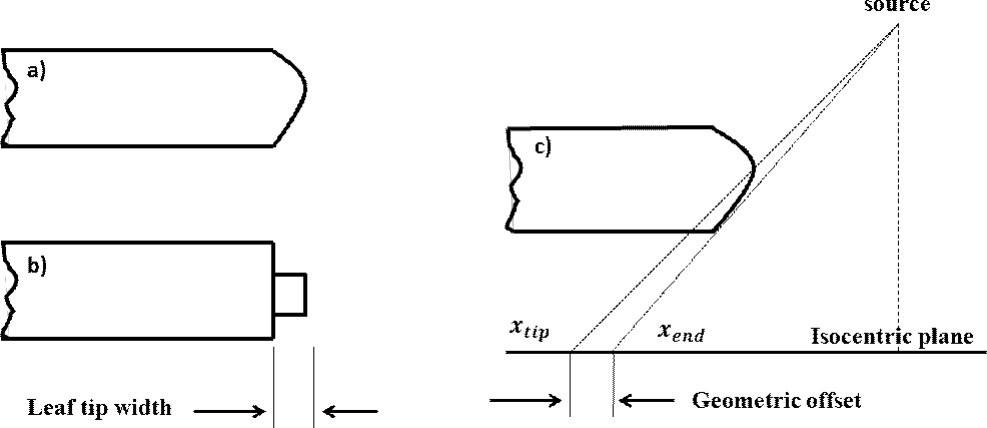
The rounded leaf tip as shown in (a) is modeled as a region with half thickness of MLC leaf as shown in (b) in RayStation TPS. The MLC leaf tip offset (c) is the difference between light projections of leaf tip $\left(x_{\_\text{tip}}\right)$ and leaf end $\left(x_{\_\text{end}}\right)$.

The MLC leaf bank is calibrated by projecting the light field on to the source‐to‐axis plane. Due to the rounded leaf end and divergence of the light field, there is a geometric offset between light projections of leaf tip ($x_{\_\text{tip}}$) and leaf end $\left(x_{\_\text{end}}\right)$, as shown in Fig. 1(c). This offset varies from 0–3 mm depending on the location of leaf tip. More details about the leaf‐tip offset have been discussed elsewhere.^12^, ^13^, ^14^ In the RayStation TPS, the plan visualization, as well as the DICOM imported/exported files, use the MLC leaf‐end position $\left(x_{\_\text{end}}\right)$ as the MLC position; the MLC leaf‐tip position ($x_{\_\text{tip}}$) is used in fluence computation. The relation between leaf‐end position and leaf‐tip position is represented using the equation:

(1) $$x_{tip}=x_{end}+offset+Gain\cdot x_{end}+Curvature\cdot x_{end}^{2}$$

For the Varian MLC, a table of geometric offset values ($x_{\_\text{tip}}-x_{\_\text{end}}$) versus MLC leaf‐end positions (−20 cm to +20 cm in 1 cm increments) is available from the vendor. This table is available in the MLC workstation in the form of a text file (MLCTABLE.TXT). The three parameters (offset, gain, and curvature) were derived by fitting the data in the table using a 2nd order polynomial.

#### A.2 Leaf‐tip width

In the RayStation planning system, the rounded leaf end is modeled as a region with one‐half the thickness of the noncurved leaf height, as shown in Figs. 1 (a) and (b). Since the leaf‐end thickness is half of the MLC leaf, the leaf‐tip radiation transmission leakage is the square root of average leaf transmission factor, which is specified in RayStation TPS. The leaf‐tip width was iteratively derived by comparing computed and measured dose off‐axis profiles of MLC defined fields at the depth of maximum dose ($d_{\text{max}}$) in water. A Scanditronix CC04 ion‐chamber (0.04 cm^3^ volume and 4 mm inner diameter) (Iba Dosimetry America, Inc., Bartlett, TN) was used to measure the dose profile in the direction parallel to MLC leaves. Six MLC defined fields were designed with different MLC leaf position [×1 cm,×2 cm]: (15,−5), (7.5,7.5),(18,0),(5,5),(−3,10),(−10,15). The negative sign represents MLC leaf travel over the central line of the radiation field. The field size perpendicular to the direction of leaf travel was maintained at 40 cm for the above measurements. Multiple sets of computed 6 MV/18 MV dose profiles were generated using different leaf‐tip width parameters (0.1 cm, 0.2 cm, 0.3 cm, 0.4 cm) while keeping other parameters, such as leaf‐tip offset, leaf radiation transmission factor, and tongue‐and‐groove width, fixed. The values of these parameters can be found in the Results section. The parameter (0.1 cm, 0.2 cm, 0.3 cm, 0.4 cm), which provided best dose‐profile matching, was selected as model parameter for leaf‐tip width.

The leaf‐tip offset and leaf‐tip width defines the radiation edge and penumbra of MLC‐shaped field. Both parameters are critical in IMRT plan dose calculation.

#### A.3 Leaf radiation transmission factor

Since IMRT usually involves the delivery of 2–10 times the number of accelerator monitor units (MUs) compared with conventional 3D plans, the leaf radiation transmission factor plays an important role in the agreement between dose calculations and measurements. In the RayStation TPS MLC model, the MLC interleaf and intraleaf leakage are modeled using the average transmission factor. The average transmission factor was iteratively derived by minimizing the difference between the calculated and measured dose distributions for five clinical IMRT plans. We tested average transmission factors ranging from 1.6% to 3.0%. For each average transmission factor, the treatment plan calculated dose was compared to MapCHECK2 (Sun Nuclear, Inc., Melbourne, FL) measurements and a secondary dose calculation (previously validated) that employs convolution/superposition using the Monte Carlo method.^(15)^ The average transmission factor that resulted in the best match between the original calculated dose and secondary measurements/calculations was selected as the model parameter.

#### A.4 Tongue‐and‐groove width

The tongue‐and‐groove structure of the leaf edge is designed to reduce the interleaf leakage. Similar to the MLC leaf tip, tongue‐and‐groove area is modeled in the RayStation TPS as a region with one‐half the thickness of leaf and is modeled using the tongue‐and‐groove width parameter. The radiation transmission through the tongue‐and‐groove region is the square root of the average MLC transmission. In this model, the tongue‐and‐groove effect is not considered when adjacent MLC leaf edges abut in RayStation TPS (i.e., it is assumed that there is no interleaf radiation leakage when MLC leaves are closed on each other). The tongue and groove is added only for the MLC leaf edge forming beam aperture in RayStation TPS (i.e., only at periphery of radiation field). Given the physical tongue‐and‐groove width of 0.04 cm and 2 mm spatial resolution of energy fluence in RayStation TPS, this parameter is expected to have a minimal effect on IMRT dose calculation.

The tongue‐and‐groove width was derived iteratively by comparing the calculated treatment plan dose with MapCHECK2 measurements and secondary dose calculations (described in the previous section) for five clinical IMRT plans. Tongue‐and‐groove widths ranging from 0.05–0.1 cm were tested, and the one resulting in the best match between the calculated treatment plan dose and measurements/secondary calculations was selected.

#### A.5 IMRT plan QA

As discussed above, the leaf radiation transmission factor and tongue‐and‐groove width were iteratively derived from clinical IMRT plan QA results. Five previously optimized clinical step‐and‐shoot and 6 MV IMRT plans (brain, locally advanced head and neck (HN), lung, pancreas, and whole pelvis) were selected to aid in optimizing the MLC model parameters. By comparing the planned dose distributions with the measured and calculated dose (via a secondary dose calculation), MLC model parameters (leaf radiation transmission factor and tongue‐and‐groove width) were iteratively adjusted. These clinical plans were initially optimized in the Pinnacle^3^ treatment planning system (Philips Radiation Oncology System, Fitchburg, WI). Plans were transferred to the RayStation planning system using the DICOM utility. Plan details are listed in Table 1. The HN and pelvis cases involved large fields, which were split due to mechanical limitations of the Varian MLC carriage (the maximum leaf‐tip distance between adjacent leaves from the same carriage may not exceed 15 cm). IMRT QA was performed via: 1) measurements using MapCHECK2, and 2) calculations using an in‐house–developed secondary convolution/superposition dose calculation algorithm that uses the Monte Carlo method. This algorithm has been previously validated and is routinely used for IMRT QA in our clinic.^(15)^ For MapCHECK2 measurements, all plan gantry angles were set 0° to avoid the angular dependence of diodes and the couch radiation attenuation. The MapCHECK2 dose calibration was performed by normalizing the output to that of a $10\times 10{\text{cm}}^{2}$ field. This reference dose measurement was performed prior to each IMRT QA measurements to account for daily linac output variations. Two gamma analysis criteria (3 mm/3% and 2 mm/2%) were applied to the comparisons between the plan and measurement, and between the plan and calculation. Only points receiving at least 10% of maximum dose were included in the gamma analysis.

**Table 1:** Five step‐and‐shoot IMRT plans (6 MV photon) used to determine MLC tongue‐and‐groove and MLC radiation transmission factors

A*natomic Site*	*Split Fields? (Y/N)*	*Field Size (cm* × *cm)*	*Number of Beams*	*Number of Segments*	*Number of MUs*	*Fraction Dose (cGy)*
Brain	N	10.5×9	7	25	176	120
Head & Neck	Y	14.5×14.5	8	41	272	120
Lung	N	12×12	6	50	359	200
Pancreas	N	12.5×13.5	7	50	429	180
Pelvis	Y	20×19.5	14	50	706	180

### B. Validation of MLC modeling parameters

A set of test cases, developed by the AAPM Task Group 119,^(6)^ was used to validate the MLC modeling parameters derived above. A CT phantom with contours provided by TG Report 119 was imported into the RayStation TPS. Five test cases (Test I1: Multitarget; Test I2: Mock prostate; Test I3: Mock head/neck; Test I4: Easier C‐shape; and Test I5: Harder C‐shape) were planned with 6 MV in the RayStation TPS using guidelines provided in the report.^(6)^ The treatment plan statistics for these plans met the plan goals, except in the case of the Harder C‐shape. However, even in this case, the deviation from plan goals was within one standard deviation (SD) of that listed in the TG Report 119. IMRT QA of the five test plans were performed using the two approaches described in the previous section. TG Report 119 only included 6 MV IMRT plans. To validate model parameters for 18 MV plans, the five 6 MV IMRT plans of test cases were converted to 18 MV plans by keeping same control points and total MU numbers. MLC model parameters were validated by comparing the treatment plan dose with MapCHECK2 measurements only (18 MV is not available in our in‐house secondary dose calculation system). Nelms et al.^(16)^ reported that the IMRT QA systematic error is more sensitive to “local” gamma analysis using dose differences relative to the local dose than conventional gamma analysis using dose difference relative to the maximum dose. As a result, we also performed local gamma analysis (3 mm/3% and 2 mm/2%) in this work.

### C. Output factors of MLC‐shaped small fields

Since IMRT involves radiation delivery via small fields, the output factors of MLC‐shaped small fields influence the accuracy of the calculated dose in IMRT plans. Sharpe et al.^(17)^ reported that the output factor is sensitive to field size deviation for small fields for both 6 MV and 18 MV photons. For a field of $1\times 1\;{\text{cm}}^{2}$, the output change of 15% was observed due to a 2 mm deviation for 6 MV and 16% for 18 MV.^(17)^ For a field of $2\times 2\;{\text{cm}}^{2}$, output change of 2% was observed due to a 2 mm deviation for 6 MV and 3% for 18 MV.^(17)^ Two MLC parameters (leaf‐tip offset and leaf‐tip width) are related to the definition of the MLC‐shaped field, which affect the calculated fluence of the MLC‐shaped field; therefore, the accuracy of these two parameters affects the output factor of MLC‐shaped field in TPS. In this project, output factors of MLC‐shaped small fields calculated in the RayStation TPS were compared to the measurements to further verify the accuracy of MLC parameters. At our institution, the output factors of MLC‐shaped small fields were measured by the Imaging and Radiation Oncology Core (IROC) on the Varian Trilogy linac (Varian Medical Systems, Palo Alto, CA) as part of the cooperative group credentialing process for National Cancer Institute (NCI)‐funded clinical trials. The MLC‐shaped small fields (cm×cm) were 2×2,3×3,4×4, and 6×6, and secondary jaw size was set at $10\times 10\;{\text{cm}}^{2}$. The output factors of small fields were measured at 10 cm effective depth and 100 SSD, and normalized to the measurement of $10\times 10\;{\text{cm}}^{2}$ field size. Detailed information of setup and measurements is available elsewhere.^(18)^ Output factors of these small fields were calculated in the RayStation planning system using a grid size of 2 mm, and compared against the IROC measurements.

## III. RESULTS

### A. MLC model parameters

**Figure 2:**
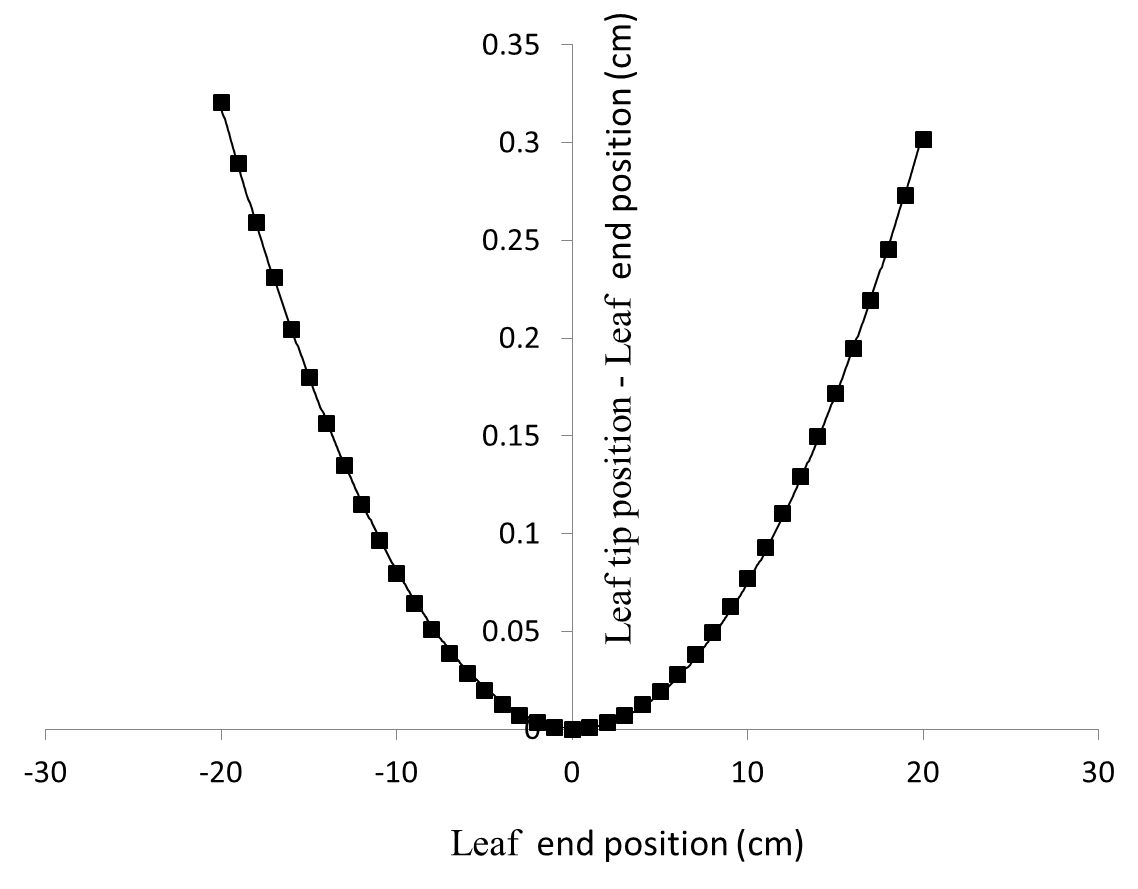
The geometric offset between light projection of MLC leaf end and projection of MLC leaf tip: ▪ indicates the data from vendor Varian, and solid line is the fitting curve ($R^{2}=0.9997$).

The geometric offset between light projection of leaf end and projection of leaf tip was modeled using equation:

(2) $$x_{tip}=x_{end}+0.0008x_{end}^{2}$$

with $R^{2}=0.9997$ (see the solid curve in Fig. 2).

**Figure 3:**
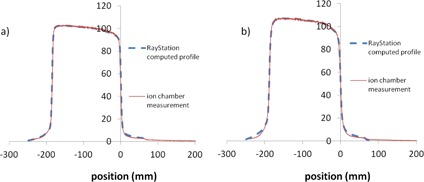
The measured profile (CC04 ion chamber) vs. RayStation‐computed profile in the MLC leaf direction for an asymmetric field 18×40 cm(X1=18 cm,X2=0) at $d_{\text{max}}$ in water for (a) 6 MV photon and (b) 18 MV photon. Both 6 MV and 18 MV photons have the same MLC parameters as following: 2.5% transmission, tongue and groove 0.05 cm, and leaf‐tip width 0.3 cm.

The off‐axis dose profile of MLC‐shaped fields computed from the RayStation planning system was compared to the measured profiles. The optimal leaf‐tip width parameter was determined to be 0.3 cm for both 6 MV and 18 MV. Figure 3 shows the comparison between computed and measured profiles for both 6 MV and 18 MV. The average MLC leaf transmission factor was 2.5%. Table 2 shows the gamma analysis results between the calculated plan dose and the measured dose with varying transmission factors for the pelvis case. While the results are similar with MLC transmission factors of 2.5 and 3.0, we chose a value of 2.5 because it is closer to that reported in previous publications.^2^, ^10^ Of note, the gamma analysis results were not as sensitive to the tongue‐and‐groove width as expected. Tongue‐and‐groove parameters of 0.05 cm and 0.1 cm were tested and no difference was found. The tongue‐and‐groove width of 0.05 cm was chosen for both 6 MV and 18 MV.

The comparison between the calculated and measured dose for the five clinical IMRT plans is shown in Table 3 (6 MV) and Table 4 (18 MV) using the following MLC model parameters: transmission factor=2.5%, tongue and groove=0.05 cm, and leaf‐tip width=0.3 cm.

**Table 2:** Gamma analysis results comparing the calculated plan dose from the TPS and the MapCHECK2 measurements for the pelvis case as a function of transmission factor. Tongue‐and‐groove width=0.05 cm, and leaf‐tip width=0.3 cm

*MLC Transmission Factor (%)*	*1.6*	*1.8*	*2.0*	*2.5*	*3.0*
Gamma Passing Rate (2 mm/2%) (%)	74.9	78.4	81.1	91.4	91.4
Gamma Passing Rate (3 mm/3%) (%)	91.8	94	95.2	98.2	98.3

**Table 3:** QA results (gamma analysis) of five clinical IMRT plans (6 MV) using MLC parameters as follows: transmission factor 2.5%, tongue and groove 0.05 cm, and leaf‐tip width 0.3 cm

	*MapCHECK2 Measurements*		*Monte Carlo Calculations*	
*Anatomic Sites*	*Passing Rate* (3%/3 mm)	*Passing Rate* (2%/2 mm)	*Passing Rate* (3%/3 mm)	*Passing Rate* (2%/2 mm)
Lung	99.9%	98.8%	99.6%	97.1%
Brain	100%	99.7%	99.8%	97.8%
HN	100%	99.0%	99.4%	96.4%
Pancreas	100%	100%	99.7%	97.2%
Pelvis (prostate)	98.2%	91.4%	98.8%	91.3%

**Table 4:** QA results (gamma analysis) of five clinical IMRT plans (18 MV) using MLC parameters as follows: transmission factor 2.5%, tongue and groove 0.05 cm, and leaf‐tip width 0.3 cm

*Anatomic Sites*	*Passing Rate* (3%/3 mm)	*Passing Rate* (2%/2 mm)
Lung	97.2%	85.5%
Brain	100%	99.0%
HN	99.2%	94.6%
Pancreas	100%	94.5%
Pelvis (prostate)	95.3%	85.5%

### B. Validation of MLC parameters

Using the above model parameters, we tested the agreement between calculation and measurements according to guidelines provided in the AAPM TG‐119 protocol. Results are summarized in Table 5 for 6 MV (the calculated plan dose from the TPS was compared with both MapCHECK2 measurements and in‐house‐developed secondary dose calculations), and for 18 MV (the calculated plan dose was compared with MapCHECK2 measurements only). For 6 MV, gamma analysis (3 mm/3%) revealed passing rates of 100% (for MapCHECK2 measurements) and >99.5% (for secondary dose calculations) in all five cases. This level of agreement surpassed the mean values (averaged across multiple institutions) reported in TG‐119. For 18 MV, gamma analysis (3 mm/3%) revealed passing rates of >97% (for MapCHECK2 measurements). TG‐119 Report did not provide IMRT results with 18 MV beams. The local gamma analysis results of MapCHECK2 measurements are summarized in Table 6

**Table 5:** QA results (gamma analysis) of five TG‐119 IMRT plans. Monte Carlo calculation is not available for 18 MV

	*MapCHECK2 Measurements (6 MV)*		*Monte Carlo Calculations (6 MV)*		*MapCHECK2 Measurements (18 MV)*	
*Anatomic Sites*	*Passing Rate* (3%/3 mm)	*Passing Rate* (2%/2 mm)	*Passing Rate* (3%/3 mm)	*Passing Rate* (2%/2 mm)	*Passing Rate* (3%/3 mm)	*Passing Rate* (2%/2 mm)
Test I1: Multitarget	100%	94.8%	100%	98.0%	100%	99%
Test I2: Mock Prostate	100%	98.5%	100%	96.2%	98.6%	91.3%
Test I3: Mock Head/neck	100%	98.5%	100%	97.5%	97.4%	90.9%
Test I4: Easy C‐shape	100%	100%	100%	98.2%	100%	100%
Test I5: Hard C‐shape	100%	94.6%	100%	97.6%	100%	99%

**Table 6:** QA results (local gamma analysis) of five TG‐119 IMRT plans measured using MapCHECK2

	*6 MV*		*18 MV*	
*Anatomic Sites*	*Passing Rate* (3%/3 mm)	*Passing Rate* (2%/2 mm)	*Passing Rate* (3%/3 mm)	*Passing Rate* (2%/2 mm)
Test I1: Multitarget	97.4%	85.5%	97.9%	96.4%
Test I2: Mock Prostate	98.5%	97.0%	98.5%	91.3%
Test I3: Mock Head/neck	99.2%	95.8%	96.1%	88.2%
Test I4: Easy C‐shape	100%	97.8%	100%	97%
Test I5: Hard C‐shape	94.5%	85.7%	96.5%	93.4%

### C. Output factors of MLC‐shaped small fields

The relative output factors of MLC‐shaped small fields of IROC measurements and RayStation calculations are shown in Table 7 for both 6 MV and 18 MV. Agreement within 2% between IROC measurement and RayStation calculation was recommended by IROC for relative output factors. The ratio of IROC measurement to RayStation calculation is within 1% for 18 MV for all fields and 2% for 6 MV, except 2 cm×2 cm field which gave 2.1% difference.

**Table 7:** Relative output factors for MLC‐defined small fields for both beam energies 6 MV and 18 MV: IROC measurements, RayStation calculations, and their ratios. The output factors were defined at 10 cm depth with 100 cm SSD, and normalized to 10 cm×10 cm field

	*6 MV*			*18 MV*		
*Field Size (cm × cm)*	*IROC*	*RayStation*	*IROC / RayStation*	*IROC*	*RayStation*	*IROC / RayStation*
10×10	1.000	1.000	1.000	1.000	1.000	1.000
6×6	0.937	0.946	0.990	0.968	0.970	0.998
4×4	0.886	0.896	0.989	0.927	0.922	1.005
3×3	0.850	0.863	0.985	0.883	0.877	1.007
2×2	0.804	0.821	0.979	0.805	0.804	1.001

## IV. DISCUSSION

### A. MLC model parameters

The purpose of this work was to investigate the optimal MLC model parameters in the RayStation TPS. Identifying feasible parameters poses a challenge because of the interdependency of these parameters on the computed dose profiles and 3D dose. The order in which these parameters are determined assumes importance. The MLC parameters with less dependence on other parameters should be determined first and the least important parameters should be last. In this work, we determined the MLC leaf‐tip offset first, followed by the MLC leaf‐tip width; the average transmission factor was determined next, and the tongue‐and‐groove parameter was determined last.

The full width of half maximum (FWHM) of dose profiles depends on the leaf‐tip width only; therefore, the FWHM was used to determine the leaf‐tip width to exclude the confounding effects of the average transmission factor. The penumbra model of MLC rounded leaf ends has been shown to be very important in step‐and‐shoot IMRT.^(3)^ It depends on both the leaf‐tip width and the average transmission factor. In the RayStation MLC model, the tongue‐and‐groove effect is not considered when opposite MLC leaf pair ends abut. The tongue and groove only affects on periphery of each segment. In addition, the computation resolution of energy fluence in RayStation is $2\times 2\;{\text{mm}}^{2}$, and the leaf tongue‐and‐groove offset is 0.4 mm, according to vendor's specification. The value of tongue and groove is not expected to have a substantial impact on step‐and‐shoot IMRT dose calculations.

Deriving the optimal beam model prior to MLC parameter optimization is critical. Matching transverse and longitudinal dose profiles (measurement and model) involves two regions in the profile: the in‐field region corresponding to the relatively flat and shoulder portion of the curve, and the out‐of‐field portion corresponding to the tail of the profile. The relatively poor agreement between measurement and TPS model calculation corresponding to the out‐of‐field portion of dose profiles has been previously reported.^19^, ^20^ While this poor agreement in the tail of the dose profile does not appreciably impact the dose accuracy in conventional 3D conventional radiation therapy (3D CRT), it assumes particular significance in IMRT, where there are many small segments and treatment delivery involves 2 to 10 times more MUs than conventional 3D CRT. As a result, small dose calculation errors outside the projection of each segment might accumulate into relatively large errors in IMRT.^(21)^ In our beam model (6 MV and 18 MV), the dose difference between measurement and calculation in the tail of the dose profile was required to be <2% of the dose at central axis. In fact, the vast majority of data points had dose differences <1%.

In this work, the MLC leaf transmission factor was not measured but iteratively derived from a comparison of measurement and calculated data from five clinical IMRT cases. The average transmission factor of 2.5% was determined to be the optimal parameter. This value is larger than the value reported in the literature^2^, ^10^ (2% for 6 MV over the clinically useful range of field size (6×6 cm to 15×15 cm) and depth (d_max_ to 20 cm)^(2)^). We attribute the larger transmission factor to the fact that the there is a larger difference between the calculated and measured dose in the out‐of‐field portion of dose profiles, as discussed above. Our beam model underestimates the out‐of‐field dose for open fields, although the error is within 2%. The higher MLC transmission factor compensates for the dose underestimation of out of field.

Our results were compared to the baseline expectations provided by AAPM Task Group 119.^(6)^ The percentage of points passing gamma criteria 3 mm/3% was better than baseline values from TG‐119 for 6 MV with MapCHECK2 measurements and Monte Carlo calculations. The test cases of 18 MV had gamma passing rate >97% (Table 5), although TG‐119 did not provide the data of 18 MV.

In AAPM TG Report 119, the gamma analysis was performed using dose differences relative to the maximum dose (global gamma or conventional gamma). Nelms et al.^(16)^ reported that, while IMRT QA errors may pass the 3 mm/3% conventional gamma analysis, they may be susceptible to regional differences between measurement and calculation. These differences are better captured using a more stringent local gamma analysis. Unsurprisingly, the QA results of local gamma analysis in Table 6 were worse than the conventional gamma analysis as shown in Table 5. The test cases involving 6 MV had local gamma passing rates (3 mm/3%) >94.5% and those involving 18 MV had passing rates >96.1%. However, 6 MV and 18 MV cases resulted in passing rates as low as 85.5% and 88.2% with the (2 mm/2%). While results have been obtained using the local gamma analysis with the 2 mm/2% criteria, there are no established baseline reference values to the best of our knowledge against which such results in this work can be benchmarked.

### B. Output factors of small MLC‐shaped field

We compared the computed output factors (in RayStation) with measurements performed by IROC at our institution on the Varian Trilogy linac. Followill et al.^(18)^ reported on small field size output factors measurements for 136 Varian‐user institutions. Our results agreed to <2%, except for the $2\times 2\;{\text{cm}}^{2}$ which revealed a difference of 2.1%. However, the agreement between measurement and calculation for the $2\times 2\;{\text{cm}}^{2}$ 6 MV output factor was superior (0.5% better) to that seen from the multi‐institution calculation average.^(18)^

## V. CONCLUSIONS

The rounded leaf end MLC model parameters in RayStation 4.0 planning system was optimized for Varian C‐series linac with a 120‐leaf Millennium MLC. For both 6 MV and 18 MV models, the optimal MLC parameters were determined to be as follows: average transmission factor=2.5%, tongue and groove=0.05 cm, and leaf‐tip width=0.3 cm. These parameters were validated using guidance from the AAPM TG‐119 protocol. IMRT QA analysis for five test cases in TG‐119 resulted in a 100% passing rate with 3 mm/3% gamma analysis criteria for 6 MV, and >97.5% for 18 MV. The IMRT commissioning of RayStation planning system is clinically acceptable. The MLC parameters reported here can serve as a reference for other RayStation and Varian users.

## ACKNOWLEDGMENTS

The authors would like thank Scott Mitchell from RaySearch Laboratories for his assistance with beam modeling. We also wish to thank our medical dosimetry colleagues Kimberly Marter, Thomas Houser, and Kristen Asbury for their assistance in IMRT planning of TG‐119 test cases.
